# The Sample Type used Affects the Levels of Gelatinases (MMP-2 and -9) and their Inhibitors (TIMP-1 and -2) in Circulating Blood of Healthy Controls and Breast Cancer Patients

**Published:** 2007-04-03

**Authors:** Kuvaja Paula, Talvensaari-Mattila Anne, Turpeenniemi-Hujanen Taina

**Affiliations:** 1 Department of Oncology and Radiotherapy; 2 Department of Obstetrics and Gynecology, Oulu University Hospital, Oulu, Finland

**Keywords:** preanalytical aspects, ELISA, gelatinases, tissue inhibitor of metalloproteinases

## Abstract

New potential tumor markers such as matrix metalloproteinases and their inhibitors have been extensively studied during the last decades. The aim is to find prognostic markers that are measurable in easily available samples, such as serum or plasma. The proper sample type to use when measuring the levels of gelatinases and their inhibitors from blood samples is currently under critical evaluation. In this study, the effect of sample type is studied in 26 healthy controls, and the result is confirmed in a series of 80 breast carcinoma patients.

Sample type had the most evident effect on the levels of TIMP-1 and MMP-9. Serum samples gave about two-fold levels of TIMP-1 compared to plasma samples (p < 0.001), with a strong linear correlation between these two (r = 0.79). Pro-MMP-9 levels were significantly affected by the presence of a blood coagulation activator in the serum sample, or a different anticoagulant in the plasma sample. The serum and plasma values had only a weak correlation (r = 0.37).

In conclusion, sample type should be carefully considered, especially when measuring proMMP-9, and plasma should be preferred for this measurement. For TIMP-1 the correlation of serum and plasma values is good; the use of serum samples can therefore be justified as long as the generally higher levels in the serum are acknowledged.

## Introduction

Matrix metalloproteinases (MMPs) form an enzyme family of zinc-dependent, structurally related enzymes that modify the extracellular matrix and regulate its turnover. Moreover, MMPs and their naturally occurring inhibitors, TIMPs, take part in complex signaling cascades affecting cell growth, tissue differentiation, angiogenesis and apoptosis. Abnormalities in these cascades cause distraction in normal functioning of the cells and tissues, leading further to diseases and even malignant transformations. Currently, a role for these enzymes has been suggested in several physiological processes requiring ECM remodeling, such as fetal differentiation of tissues and wound healing. In addition, these enzymes have been studied in a variety of pathological conditions including cardiovascular diseases, rheumatic diseases, and malignancies (reviewed in Sternlicht and Werb, 2001; Turpeenniemi-Hujanen, 2005). The tumor tissue expression of the gelatinases MMP-2 and -9 has been shown to correlate with poor prognosis in several malignancies (Turpeenniemi-Hujanen, 2005). Some recent studies suggest that tissue inhibitor of metalloproteinases-1 (TIMP-1) and gelatinase B (MMP-9) could be utilized as novel tumor markers in the determination of prognosis in the carcinomas of head and neck, breast, ovary, and colon (Holten-Andersen et al. 2000; Ranuncolo et al. 2003; Talvensaari-Mattila et al. 2005a; Talvensaari-Mattila et al. 2005b; Rauvala et al. 2005; Ruokolainen et al. 2005a; Ruokolainen et al. 2005b). In these studies, TIMP-1 and MMP-9 levels are measured from circulating blood; however, the sample type used varies. Since MMPs and TIMPs have influence in several normal processes, the physiological and preanalytical factors affecting these enzyme levels should be carefully identified in order to use these enzymes as tumor biomarkers. Platelets physiologically contain MMP-9 and TIMP-1, and the use of serum has therefore been questioned in tumor marker studies (Jung et al. 2001; Jung, 2005). However, other factors such as coagulation accelerators or anticoagulants could also have an effect on these enzyme levels.

The aims of this study were firstly, to determine whether the sample type (serum or plasma) used affects the levels of gelatinases and their inhibitors measured in blood. This experiment was done on 26 healthy volunteers using 4 different sample types. Secondly, the differences between serum and plasma levels of metalloproteinases and their inhibitors were studied in the preoperative blood of 80 breast cancer patients in order to verify that the observations on sample type apply to patient material. Thirdly, the levels of gelatinases and their inhibitors in the blood of healthy controls and patients were compared.

## Materials and Methods

### Study design and sample collection

The material consisted of blood samples from 80 patients diagnosed with primary breast cancer in Oulu University hospital during March 2003–March 2004, and 26 healthy controls volunteering for the project. All patients and controls in this study have received and signed an informed consent, where they give permission to use their blood samples for research purposes.

Healthy controls gave 2 different serum and 2 different plasma samples. The serum samples from each volunteer were collected into one glass tube (367608) that contained no artificial coagulation activator, and into another plastic serum tube with silicone-coated interior and gel, with added artificial coagulation activator, referred to as “serum+” in the text (367957). Plasma samples were collected into one lithium-heparin plastic tube (367374), and into another plastic tube containing K_2_EDTA (368856). Patient samples were collected into one serum tube corresponding to serum+, and one EDTA plasma tube. All sample tubes were purchased from BD Vacutainer systems, Plymouth, U.K. The patient samples were collected during March 2003–March 2004, and control samples during September 2005.

After collecting, the blood samples were allowed to clot thoroughly for 30 minutes before centrifugation, centrifuged at 3000 rpm for 10 minutes, and serum/plasma was aspirated into polypropylene micro tubes (Sarstedt, Nurnberg, Germany), and stored in a refrigerator at −75 °C.

TIMP-1, TIMP-2, proMMP-9, and proMMP-2/ TIMP-2 complex concentrations were determined from all samples. In addition, total proMMP-2 and active MMP-2 concentrations were determined from 20 control samples.

The descriptive histopathological and clinical data of breast carcinoma patients is summarized in Table 3.

### ELISA analysis

The enzyme-linked immunosorbent assay (ELISA) was used to detect the circulating TIMP-1, TIMP-2, proMMP-9, proMMP-2 levels, active MMP-2 levels, and proMMP2-TIMP2 complex levels from serum and plasma samples. TIMP-1, TIMP-2, proMMP-9 and proMMP2-TIMP2 complex were detected by coating 8-well E.I.A/R.I.A strips for microtiter plates (Corning Inc., Corning, NY, U.S.A.) with monoclonal antibodies, anti-TIMP-1 (DB120D1), antiMMP-9 (Ge-213), or anti-TIMP-2 antibody (for TIMP-2 and proMMP-2/TIMP-2 complex, clone T2-101). The diluted serum or plasma samples were then added, followed by polyclonal anti-TIMP-1, anti-TIMP-2 (DB-205), anti-MMP-9 (DB-209), or anti-MMP-2 (for proMMP-2/TIMP-2 complex, DB-202) as the second antibody (all antibodies described in these ELISA analyses were purchased from SBA Sciences, Oulu, Finland). Anti-chicken horseradish peroxidase enzyme (Chemicon International, Temecula, CA, U.S.A.) served as the enzyme conjugate, and the reaction was visualized by OPD (o-phenylenediamine dihydrochloride) enzyme substrate (Sigma, Steinheim, Germany). The absorbances were read at 492 nm wavelength by Anthos Reader 2001.

A commercial assay kit (Human Biotrak Elisa system for detecting MMP-2 by Amersham Biosciences, Buckinghamshire, England) was used to detect the total and active MMP-2 levels. The assay for total proMMP-2 (RPN 2617) recognized both free proMMP-2 and proMMP2-TIMP2 complexes, whereas the assay used in measuring the active MMP-2 (RPN 2631) recognized only free active forms of MMP-2. The assay was conducted following the manufacturer’s instructions.

Each sample was run in duplicate in order to minimize intra-assay variation. The absorbance values for standard samples and the standard curves constructed for each assay were compared and used to minimize the interassay variation. The sensitivity of the assays was 1ng/ml for TIMP-1 and MMP-9, and 2 ng/ml for TIMP-2 and the proMMP-2/TIMP-2 complex. Sensitivities for the total proMMP-2 and active MMP-2 were 0.37 ng/ml and 190 pg/ml, respectively.

### Statistical analysis

The distributions of the serum and plasma concentrations were first tested for normality for each protein in the group of patients and controls separately. Normality of the distribution was determined by Kolmogorow-Smirnov’s test with Lillefors significance correction. Descriptive statistics (mean, median) are given according to normality of the distribution. In the control group, one-way ANOVA analysis with Scheffe’s test for subset analysis or Kruskall-Wallis test were first used in the analysis of 4 different sample types. In the case of significant results, the analyses were continued by pairing the variables and analyzing them with Student’s T-test or Mann-Whitney’s U-test. T-test and U-test were used in analyzing the differences between patients’ serum and plasma samples and between patients and controls. The linearity of the correlation of serum and plasma concentrations was tested with Pearson r and linear regression model. P-values < 0.05 were considered significant. All statistics were performed using SPSS software.

## Results

### Sample type effect in healthy controls

The effect of the sample type used was first studied in the group of twenty-six healthy volunteers. Sample type was found to affect both MMP and TIMP concentrations. Significant differences were observed in the assays for TIMP-1, proMMP2-TIMP2 complex, proMMP-9 and active MMP-2.

**Figure 1:**
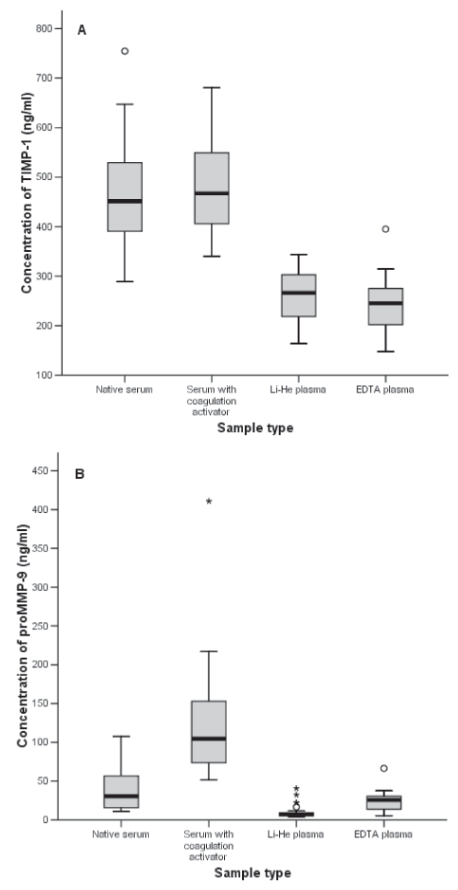
Protein concentrations according to sample type for **A**) TIMP-1, **B**) proMMP-9 in healthy controls (n = 26).

For TIMP-1, the plasma levels were significantly lower than the serum levels (p < 0.001, Table 1). The subsets of native serum, serum with coagulation activator, LiHe and EDTA plasma were examined by Scheffe’s test. Coagulation activation in the serum samples and the type of anticoagulant used in the plasma samples did not affect the TIMP-1 levels, resulting in native serum paired with serum+, and LiHe plasma paired with EDTA plasma as homogeneous subsets (Fig 1A).

ProMMP-9 serum levels were significantly affected by coagulation activation, giving the median 30.4 ng/ml for native serum and the mean 124.8 ng/ml for serum+ (p < 0.001, Table 1, Fig 1B). ProMMP-9 levels were also significantly affected by anticoagulant type, with lower MMP-9 levels for LiHe plasma than for EDTA plasma (p < 0.001, Fig 1B). Subsets of different sample types were then paired for comparison, and studied by T-test or U-test. The proMMP-9 levels in the lithium- heparin plasma, and serum samples with coagulation activator (serum+) differed signifi-cantly from all other sample types, giving p < 0.001 in all subgroup analyses of 2 variables (Fig 1B, data not shown). When EDTA plasma and native serum were paired for comparison, no significant differences in the proMMP-9 levels were found in the U-test (p = 0.06, Fig 1B, data not shown).

For the proMMP2-TIMP2 complex, the protein concentrations in the EDTA plasma were lower than in other sample types (p < 0.001, Table 1). For active MMP-2, LiHe plasma levels were significantly lower than the corresponding serum levels (Table 1). The coagulation activator did not have any effect on the serum levels for active MMP-2 and the proMMP2-TIMP2 complex. For TIMP-2 and proMMP-2, no significant differences were found between the sample types (Table 1).

### Effect of sample type in breast cancer patients

**Figure 2:**
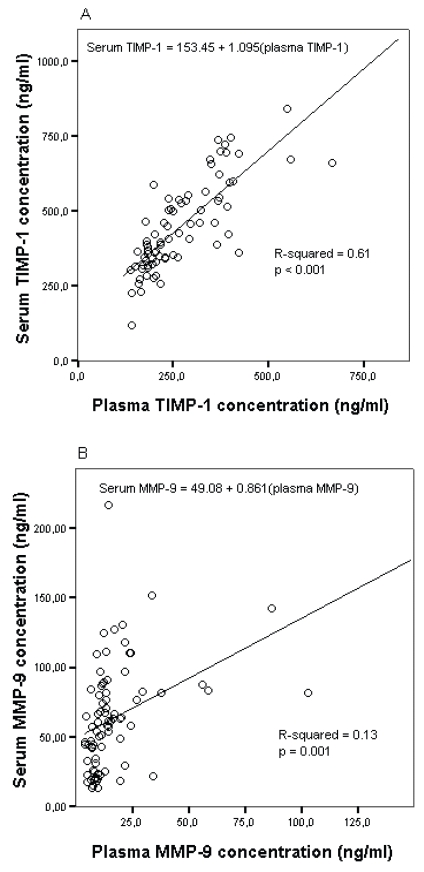

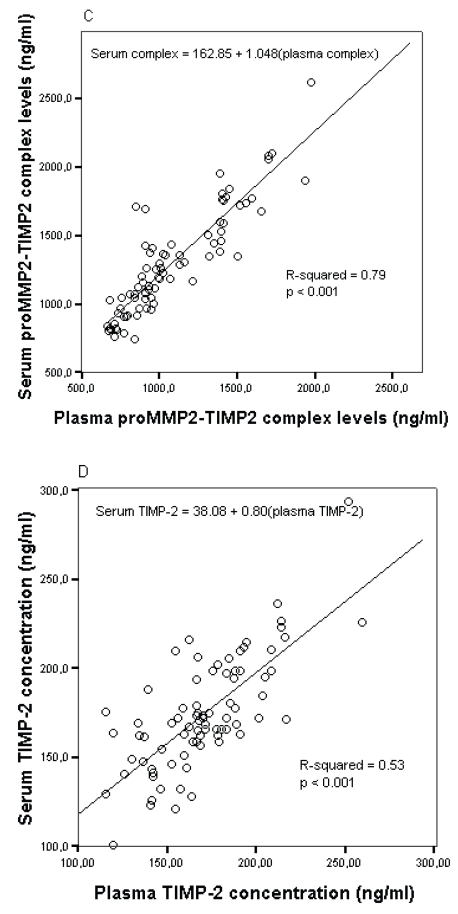
Linear regression models for serum **A)** TIMP-1, **B)** MMP-9, **C)** proMMP2-TIMP2 complex, **D)** TIMP-2 in breast cancer patient material.

Similar effects caused by sample type were discovered when blood samples of 80 breast cancer patients were analyzed. For these samples, the levels of TIMP-1, proMMP-9, proMMP2-TIMP2 complex and TIMP-2 were compared in serum with coagulation activator (serum+) and in EDTA plasma. TIMP-1 levels were significantly lower in plasma (median 237.8 ng/ml) than in serum (median 408.4 ng/ml) (p < 0.001, Table 2). Although the levels were different, the ranges were overlapping and there was a strong linear correlation between plasma and serum concentrations with Pearson r = 0.79 and R-squared 0.61, the linear regression analysis model of plasma TIMP-1 explaining 61% of the total variation of serum TIMP-1 values (Table 2, Fig 2A). For the proMMP2-TIMP2 complex, the levels were significantly lower in EDTA plasma (median 960.9 ng/ml) than in serum (mean 1296.9 ng/ml) samples (p < 0.001, Table 2). Despite the differences in the mean/median levels, there was a strong linear correlation between plasma and serum values with Pearson r = 0.89 and R-squared 0.79 (Table 2, Fig 2C). ProMMP-9 concentrations were found to be significantly lower in plasma samples than in serum+ samples (p < 0.001, Table 2). There was a weak linear correlation between plasma and serum concentrations, but the linear regression model of plasma TIMP-1 explained only 13% of the total variation of serum TIMP-1 values (Table 2, Fig 2B). For TIMP-2 no differences were found between plasma and serum samples, and there was a strong linear correlation between serum and plasma TIMP-2, the linear regression model explaining 53% of the total variation of serum TIMP-2 values (Table 2, Fig 2D).

### Differences between patients and controls

**Figure 3:**
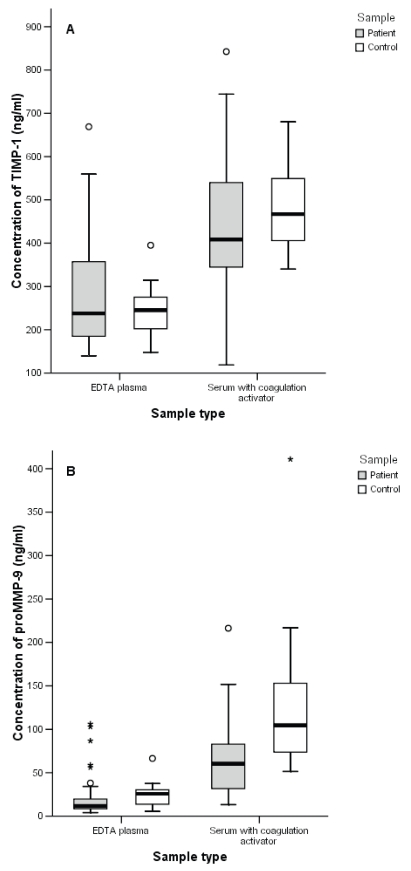
Corresponding protein concentrations according to sample type for **A)** TIMP-1 and **B)** proMMP-9 in breast carcinoma patients and healthy controls.

The ranges for TIMP-1 concentrations were wider in both plasma and serum samples of breast cancer patients compared with healthy controls (Fig 3A), although there were no significant differences between patients and controls for TIMP-1 in plasma or serum. ProMMP-9 concentrations in both plasma (p = 0.002) and serum (p < 0.001) were significantly lower in the blood of breast cancer patients than in control samples (Fig 3B). The protein concentrations in both plasma (p < 0.001) and serum (p < 0.001) were also lower for TIMP-2 in patient samples compared with controls (data not shown). For the proMMP-2/TIMP-2 complex no significant differences were found between patients and controls (data not shown).

## Discussion

In this study, sample type was found to have an effect on the concentrations of metalloproteinases and their inhibitors in circulating blood. Sample type had the clearest effect on the levels of proMMP-9 and TIMP-1. Platelets contain both MMP-9 and TIMP-1, and it has been shown that platelet aggregation during clotting can lead to increased release of MMP-9 and TIMP-1 (Holten-Andersen et al. 2002; Sheu et al. 2004).

Recent studies suggest that TIMP-1 has prognostic value in head and neck SCC (Ruokolainen et al. 2005a), colorectal carcinoma (Holten-Andersen et al. 2000), ovarian carcinoma (Rauvala et al. 2005) and breast carcinoma (Talvensaari-Mattila et al. 2005a). Some of these results are based on serum (Talvensaari-Mattila et al. 2005a; Ruokolainen et al. 2005a; Rauvala et al. 2005) and some on plasma samples (Holten-Andersen et al. 2000). The use of serum has been questioned because of the generally higher levels of serum TIMP-1 in comparison with plasma TIMP-1 (Jung et al. 2005). In an experiment by Jung et al. (2005), up to 5–7 times higher concentrations of TIMP-1 were shown in serum versus plasma. The blood samples in this experiment were collected into serum tubes that contained artificial coagulation activators. However, in our experiment the levels of TIMP-1 in the serum were about 2-fold higher than in the plasma in patients and in controls, and were not affected by coagulation activators or anticoagulants. In addition, a strong correlation existed between TIMP-1 in serum and plasma samples. Patients and controls did not have significant differences in their serum or plasma TIMP-1 levels, although the ranges for serum and plasma TIMP-1 were wider in patients in comparison with controls. This could indicate the presence of a disturbance, such as cancer, that could affect the levels of the analytes.

Most studies on the effect of preanalytical conditions on metalloproteinase concentrations in the blood concern the analysis of proMMP-9. It has been shown in several studies that serum has generally higher levels of MMP-9 than do plasma samples. This has been documented using both ELISA and gelatin zymography (Jung, 2005; Jung et al. 2001; Gerlach et al. 2005; Makowski et al. 2003; Mannello, 2003a; Mannello et al. 2003b). In our experiment it was evident that coagulation activators had an effect on the proMMP-9 serum levels, giving up to 4-fold MMP-9 levels in comparison with native serum, probably due to platelet release of MMP-9. A similar observation was made by Jung et al. (2001). However, it is notable that in the experiment by Jung et al. (2001) the native serum with no coagulation activator had markedly higher MMP-9 levels than what was measured in plasma. In our experiment, the native serum concentrations of MMP-9 were found to resemble those measured in EDTA plasma. Anticoagulant type also had a significant effect, giving LiHe plasma significantly lower concentrations of MMP-9.

The use of proper sample type in measuring MMP-9 from blood is discussed in many studies, and some show that citrate plasma might be the safest option (Mannello, 2003a; Mannello et al. 2003b), suggesting that serum should not be used at all in these measurements. However, in recent studies done on serum, using material collected into native serum tubes, proMMP-9 has been shown to have prognostic value in breast carcinoma and head and neck SCC (Talvensaari-Mattila et al. 2005b; Ruokolainen et al. 2005b). In the study by Talvensaari-Mattila et al. (2005b), low preoperative serum proMMP-9 was found to correlate with poor relapse-free survival. In our current study, breast cancer patients were found to have lower serum and plasma proMMP-9 concentrations than healthy controls, the difference being even clearer in serum samples. This might suggest that higher serum proMMP-9 could not only be due to artefact caused by platelet release of MMP-9, but that MMP-9 measured in the serum actually relates to disease progression. Therefore, proMMP-9 measurements done on serum could still have some value, even though caution should be maintained in interpreting the results. It is notable that the plasma proMMP-9 concentration could only explain 13% of the total variation in the serum proMMP-9 values in this study (when a coagulation activator was present), and the use of plasma samples is therefore probably a safer option to produce reliable results.

The blood samples of breast carcinoma patients were exposed to a longer storage time than control samples in this study. It has been shown by Rouy et al. (2005) that detectable levels of proMMP-9 decrease heavily in citrate plasma samples over time. Therefore we cannot exclude that the higher levels of control samples might be partly due to enzyme degradation in this study. The effect appears to be specific for proMMP-9, since no such differences were found for proMMP-2 or TIMP-1 (Rouy et al. 2005). Since proMMP-9 is sensitive for several (coagulation activators, anticoagulants, storage time) preanalytical issues, standardization is crucial if this enzyme is to be measured from circulation.

For the proMMP2-TIMP2 complex the use of EDTA plasma as a sample gave lower concentrations than did other sample types. It has previously been suggested that the use of EDTA in blood samples might alter the measured MMP levels, since EDTA is able to chelate Zn2^+^, possibly leading to lower measured proMMP-2 levels (Imafuku et al. 2002), and in some studies (Jung et al. 1998; Mannello et al. 2003b) lower levels of proMMP-2 immunoreactive protein have been observed in EDTA plasma. It has been shown that during aggregation, platelets release MMP-2 in its latent form (Sawicki et al. 1997) as well as other components of the proMMP2/MT1-MMP/TIMP-2 system (Kazes et al. 2000). However, we did not observe differences in the total proMMP-2 concentrations between different sample types, but when measuring active MMP-2 the LiHe plasma gave a lower concentration than serum samples, suggesting platelet release of MMP-2.

In a study by Jung et al. (1998), high concentrations of TIMP-2 were also found in heparin plasma in comparison with EDTA plasma and serum. In the current study no such differences were found. Instead, we found surprisingly that breast cancer patients had significantly lower TIMP-2 levels than did healthy controls. In breast carcinoma, no evidence currently exists that TIMP-2 could be prognostic when measured in peripheral blood. However, the presence of the disease might alter the balance of the proteolysis. Low TIMP-2 levels in the blood of breast carcinoma patients could indicate more activated MMP-2 and therefore higher usage of TIMP-2, or alternatively, the lower levels could be due to lower production of TIMP-2, leading to lesser inhibition of MMP-2 activity.

Taken together, blood sample type used was found to have a significant effect on metalloproteinases and their inhibitor levels. The results on the sample type effect obtained using healthy controls were reproducible in patient material, although the levels of the studied enzymes showed some differences between patients and controls.

The blood sample type had the clearest effect when measuring MMP-9 or TIMP-1. MMP-9 levels are affected by coagulation activation and the anticoagulant used, and MMP-9 is therefore more safely determinable in plasma samples. TIMP-1 gives higher serum than plasma levels, but the correlation between these values is very good. Serum might be a valid sample choice for measuring TIMP-1 as long as the generally higher levels are acknowledged.

## Figures and Tables

**Table 1:** Protein concentrations according to sample type in healthy controls.

Protein	Sample type *	Mean/median (ng/ml)	Range (ng/ml)	Significance
TIMP-1				
	Native serum	462.7	288.9–754.6	
	Serum +	486.5	340.1–680.3	
	LiHe	259.7	163.7–344.0	
	EDTA	243.9	147.9–395.1	p < 0.001
TIMP-2				
	Native serum	201.8	144.0–311.1	
	Serum +	197.7	167.1–317.2	
	LiHe	187.2	125.6–347.3	
	EDTA	199.8	141.1–307.1	N. S
MMP2-TIMP2				
	Native serum	1456.4	749.8–2168.7	
	Serum +	1436.7	805.2–2087.7	
	LiHe	1384.2	854.8–2198.3	
	EDTA	1119.1	641.6–1780.6	p < 0.001
ProMMP-9				
	Native serum	30.4	10.7–107.3	
	Serum +	124.8	51.4–410.9	
	LiHe	7.3	3.8–40.6	
	EDTA	24.3	5.5–66.5	p < 0.001
ProMMP-2				
	Native serum	1259.9	793.9–1881.9	
	Serum +	1197.3	777.3–1790.3	
	LiHe	1224.7	783.0–1894.2	
	EDTA	1195.5	770.6–1791.2	N. S
Active MMP-2				
	Native serum	29.4	10.8–68.1	
	Serum +	33.5	10.0–78.7	
	LiHe	8.51	5.67–14.7	p < 0.001

* Serum + indicating serum with coagulation activator.

**Table 2:** Protein concentrations according to sample type in breast carcinoma patients.

Protein	Sample type	Mean/median (ng/ml)	Range (ng/ml)	p-value*	Pearson r	R-squared
TIMP-1						
	Serum +	408.4	118.6–842.5			
	EDTA	237.8	139.8–669.0	p < 0.001	0.79	0.61
TIMP-2						
	Serum +	170.4	100.5–293.8			
	EDTA	170.8	115.6–259.8	N.S.	0.73	0.53
MMP2-TIMP-2						
	Serum +	1296.9	743.0–2616.4			
	EDTA	960.9	662.7–1975.2	p < 0.001	0.89	0.79
MMP-9						
	Serum +	63.3	13.3–216.4			
	EDTA	11.8	4.0–106.2	p < 0.001	0.37	0.13

* Significance of the sample type effect (plasma/serum).

**Table 3:** Clinico-pathological parameters and patient characteristics of breast carcinoma patients.

Tumor parameter	n	% of patients	Patient characteristics	n	% of patients	median (range)
Histological type						
ductal	57	71.3	Menopausal status			
lobular	13	16.3	pre	26	35.1	
DC in situ	4	5.0	post	48	64.9	
tubular	3	3.8	Surgery			
papillar	1	1.3	mastectomy	38	47.5	
mucinous	1	1.3	breast			
Size of the tumor			conserving	42	52.5	
<2cm	51	66.2	Chemotherapy			
2–5cm	24	31.2	yes	31	38.8	
>5cm	2	2.6	no	49	61.3	
Nodal status			Radiotherapy			
negative	50	62.5	yes	70	87.5	
≤2 positive nodes	19	23.8	no	10	12.5	
>2 positive nodes	11	13.8	Endocrine			
Stage*			therapy			
1	36	47.4	yes	30	37.5	
2A	23	30.3	no	50	62.5	
2B	16	21.5	Age at diagnosis			56 (28–87)
3A	1	1.3				
Histological grade**						
1	9	12.3				
2	35	47.9				
3	29	39.7				
Hormone receptor status						
ER positive	61	81.3				
ER negative	14	18.7				
PR positive	45	60.0				
PR negative	30	40.0				

* according to UICC TNM classification.
